# Comparison of Intake and Systemic Relative Effect Potencies of Dioxin-like Compounds in Female Mice after a Single Oral Dose

**DOI:** 10.1289/ehp.1206336

**Published:** 2013-05-03

**Authors:** Karin I. van Ede, Patrik L. Andersson, Konrad P.J. Gaisch, Martin van den Berg, Majorie B.M. van Duursen

**Affiliations:** 1 Institute for Risk Assessment Sciences, Utrecht University, Utrecht, the Netherlands; 2 Department of Chemistry, Umeå University, Umeå, Sweden

**Keywords:** dibenzofurans, dioxins, PCBs, PCDDs, PCDFs, polychlorinated biphenyls, systemic REPs, TEF concept

## Abstract

Background: Risk assessment for mixtures of polychlorinated dibenzo-*p*-dioxins (PCDDs), polychlorinated dibenzofurans (PCDFs), and polychlorinated biphenyls (PCBs) is performed using the toxic equivalency factor (TEF) approach. These TEF values are derived mainly from relative effect potencies (REPs) linking an administered dose to an *in vivo* toxic or biological effect, resulting in “intake” TEFs. At present, there is insufficient data available to conclude that intake TEFs are also applicable for systemic concentrations (e.g., blood and tissues).

Objective: We compared intake and systemic REPs of 1,2,3,7,8-pentachlorodibenzodioxin (PeCDD), 2,3,4,7,8-pentachlorodibenzofuran (4-PeCDF), 3,3´,4,4´,5-pentachlorobiphenyl (PCB-126), 2,3´,4,4´,5-pentachlorobiphenyl (PCB-118), and 2,3,3´,4,4´,5-hexachlorobiphenyl (PCB-156) in female C57BL/6 mice 3 days after a single oral dose.

Methods: We calculated intake REPs and systemic REPs based on administered dose and liver, adipose, or plasma concentrations relative to TCDD. Hepatic cytochrome P450 1A1–associated ethoxyresorufin-*O*-deethylase (EROD) activity and gene expression of *Cyp1a1*, *1a2* and *1b1* in the liver and peripheral blood lymphocytes (PBLs) were used as biological end points.

Results: We observed up to one order of magnitude difference between intake REPs and systemic REPs. Two different patterns were discerned. Compared with intake REPs, systemic REPs based on plasma or adipose levels were higher for PeCDD, 4-PeCDF, and PCB-126 but lower for the mono*-ortho* PCBs 118 and 156.

Conclusions: Based on these mouse data, the comparison between intake REPs and systemic REPs reveals significant congener-specific differences that warrants the development of systemic TEFs to calculate toxic equivalents (TEQs) in blood and body tissues.

Polychlorinated dibenzo-*p*-dioxins (PCDDs), polychlorinated dibenzofurans (PCDFs), and polychlorinated biphenyls (PCBs) are persistent and widespread contaminants. Of the 419 possible congeners that exist, 7 PCDDs, 10 PCDFs, and 12 non-*ortho* and mono-*ortho* PCBs are classified as having dioxin-like effects. Most, if not all, toxic effects of dioxin-like compounds (DLCs) are mediated through the aryl hydrocarbon receptor (AHR); the toxic effects of these DLCs include endocrine, developmental, immune, and carcinogenic effects, among others (Birnbaum 1994; Birnbaum and Tuomisto 2000; Safe 1990; White and Birnbaum 2009). Humans are exposed to a complex mixture of these DLCs mainly through the diet, with food of animal origin being the most important source. Although exposure has significantly decreased during the past decades (De Mul et al. 2008; Fürst 2006), current human exposure is still above the tolerable daily intake (TDI) or reference dose (RfD) levels for parts of the population in some countries (Bilau et al. 2008; De Mul et al. 2008; Llobet et al. 2008; Loutfy et al. 2006; Tard et al. 2007). Therefore, improving the risk assessment process for this class of compounds remains important and societally relevant.

Currently, risk assessment of DLCs is based on the toxic equivalency factor (TEF) approach (Safe 1990, 1994) endorsed by the World Health Organization (WHO) (Van den Berg et al. 1998, 2006). Each congener-specific TEF is derived from multiple relative effect potencies (REPs) determined from a range of AhR-specific end points [e.g., cytochrome P450 1A1 (CYP1A1) activity]. The toxic or biological potency of a congener is compared to that of 2,3,7,8-tetrachlorodibenzo-*p*-dioxin (TCDD). A shortcoming of the TEF concept originates from the fact that the TEFs were established primarily from *in vivo* end points linking administered dose levels (via oral exposure) to toxic or biological effects, resulting in “intake” TEFs (^intake^TEFs). Consequently, these ^intake^TEFs are applicable only for situations in which ingestion (e.g., food intake, consumption of breast milk) is known. However, because ingestion data for humans is often lacking or difficult to establish, blood or adipose tissue levels are frequently used to quantify the relative exposure to humans. Subsequently, regulatory authorities commonly calculate risks based on blood or adipose tissue (systemic) levels using these ^intake^TEFs. Unfortunately, even for the most relevant DLCs, experimental validation is in sufficient to either reject or accept this application of ^intake^TEFs for blood or tissue levels. There is limited evidence suggesting that the use of ^intake^TEFs instead of ^systemic^TEFs may lead to inaccurate interpretation of the risk because of congener-specific toxicokinetic differences (Chen et al. 2001; DeVito et al. 1998; Hamm et al. 2003). Properties such as absorption, distribution, metabolism, and excretion can clearly contribute to the potency of a congener (Budinsky et al. 2006; DeVito and Birnbaum 1995; DeVito et al. 1997, 2000) and may be misinterpreted when relying solely on ^intake^TEFs. At the most recent WHO expert meeting (in 2005) where the TEFs were (re)evaluated, it was concluded that insufficient data were available to develop ^systemic^TEFs, leaving a major gap in the risk assessment process for DLCs (Van den Berg et al. 2006). To fill this data gap, the European Union (EU) project SYSTEQ was initiated, with the main objectives of establishing *in vivo* ^systemic^REPs in the mouse and rat, with special focus on effects in peripheral blood lymphocytes (PBLs) as potential biomarkers of exposure.

In the present study we compared ^intake^REPs and ^systemic^REPs in female C57BL/6 mice based on the administered dose and liver, adipose, or plasma concentrations. We used 2,3,7,8-tetrachlorodibenzodioxin (TCDD), 1,2,3,7,8-pentachlorodibenzodioxin (PeCDD), 2,3,4,7,8-pentachlorodibenzofuran (4-PeCDF), 3,3´,4,4´,5-pentachlorobiphenyl (PCB-126), 2,3´,4,4´,5-pentachlorobiphenyl (PCB-118), and 2,3,3´,4,4´,5-hexachlorobiphenyl (PCB-156), which represent approximately 90% of the dioxin-like activity in the human food chain (Liem et al. 2000); we also included the non-dioxin-like 2,2´,4,4´,5,5´-hexachlorobiphenyl (PCB-153). Three days after exposure, we calculated ^intake^REPs and ^systemic^REPs for hepatic CYP1A1-associated ethoxyresorufin-*O*-deethylase (EROD) activity and *Cyp1a1*, *1a2*, and *1b1* gene expression in the mouse liver and PBLs.

## Materials and Methods

*Chemicals*. TCDD, PeCDD, 4-PeCDF, and PCB-126 were purchased from Wellington Laboratories Inc. (Guelph, Ontario, Canada) and dissolved in corn oil (ACH Food Companies Inc., Oakbrook, IL, USA); concentrations were then checked and confirmed by Wellington Laboratories Inc. We purchased PCB-118, PCB-156, and PCB-153 from Cerilliant Corp. (Round Rock, TX, USA). These three PCBs and corn oil (Sigma-Aldrich, Stockholm, Sweden) were purity checked; and PCB-118 and PCB-156 were purified at the Department of Chemistry, Umeå University. Before purification, PCB-118 contained 85 ng toxic equivalents (TEQ)/g and PCB-156 contained 201 ng TEQ/g. The final toxic equivalent (TEQ) contributions of impurities were 6.6 ng TEQ/g (PCB-118), 36 ng TEQ/g (PCB-156), and 0.41 ng TEQ/g (PCB-153), levels we considered to have no influence on the final outcome of our results. PCBs were dissolved in corn oil after purification. All tested congeners were further diluted in corn oil (Sigma-Aldrich) at the Institute for Risk Assessment Sciences, Utrecht University).

*Animals*. Eight-week-old female C57BL/6 mice (Harlan laboratories, Venray, the Netherlands) were randomly assigned to treatment groups (six animals per group) and allowed to acclimate for 1.5 weeks. The animals were housed in groups in standard cages and conditions (23 ± 2°C, 50–60% relative humidity, 12-hr dark/light cycle) with free access to food and water. Mice received a single dose of test compound by oral gavage at a dosing volume of 10 mL/kg body weight (BW). Mice treated with corn oil vehicle (10 mL/kg BW) served as controls. For each congener, five different doses were administered, ranging from 0.5–100 μg/kg BW (TCDD) to 5,000–500,000 µg/kg BW (PCB-153). Detailed information on doses is provided in Supplemental Material, Table S1 (http://dx.doi.org/10.1289/ehp.1206336). On day 3 after dosing, animals were euthanized by CO_2_/O_2_ asphyxiation, and blood was immediately collected from the abdominal aorta. The liver, thymus, spleen, and adipose tissue were removed, weighed (liver, thymus, and spleen), snap frozen, and stored at –80°C. All animal treatments were performed with permission of the Animal Ethical Committee (DEC Utrecht) and performed according to the Law on Animal Experiments (1977). Animals were treated humanely and with regard for alleviation of suffering.

*Compound analysis*. Adipose and liver tissues samples were homogenized in Na_2_SO_4_, followed by extraction and clean-up in one step, and then eluted with 200 mL 1:1 hexane:dichloromethane on an open column packed with 40% wt/wt H_2_SO_4_-impregnated silica and KOH-silica. Blood plasma samples were extracted on an open column using Chem-Elut (Agilent Technologies, Santa Clara, CA, USA) and then NaCl eluted with 75 mL 3:2 hexane:2-propanol. Clean-up was performed using a miniaturized silica column (as described above), and samples were eluted using 30 mL hexane. Because the samples typically contained high levels of the analytes, only a small fraction was evaporated and analyzed. Prior to evaporation, we spiked a fraction of the samples with ^13^C-labeled standards. We checked potential loss of analytes during extraction and clean-up by reextracting the samples using the identical protocol used for the samples. This procedure indicated that the losses that occurred during this first step were minor, and thus most likely do not significantly contribute to the measured outcomes. Tetradecane was added prior to evaporation. Sample analysis followed the U.S. Environmental Protection Agency Method 1613 (U.S. Environmental Protection Agency 1994) using single ion monitoring mode on an Agilent 6809N gas chromatograph (Agilent Technologies) coupled with a Micromass Ultima Autospec Ultra high-resolution mass spectrometer (HRMS; Waters Corp., Milford, MA, USA). Compounds were separated on a 60 m × 0.25 mm DB5-MS column (0.25 μM; J&W Scientific, Folsom, CA, USA). The HRMS was operated with electron impact ionization with electron energy of 35 eV and an ion source temperature of 250°C. To reduce the number of analyses, samples were pooled before clean-up. To retain unique individual results, liver, adipose, and plasma samples were not pooled within the same treatment group of one congener, but between similar exposure levels of TCDD, PeCDD, 4-PeCDF, and PCB-126 or PCB-118, PCB-156, and PCB-153. This method was used because full congener–specific separation could be achieved on the high-resolution GC–HRMS. For lipid determination, samples were evaporated to dryness after the extraction step, and the amount of lipids was determined gravimetrically. Concentrations were calculated based on lipid weight and wet weight. The analysis of samples for the PCB-118 5,000 μg/kg BW dose failed during the procedure; thus analysis for this group could not be completed.

*Plasma and PBL isolation*. Blood from two mice was pooled (total volume of approximately 1.4 mL); plasma and PBLs were then isolated using Ficoll Paque gradient (GE Healthcare Europe, Diegem, Belgium). Plasma samples were stored at –80°C until compound analysis. Isolated lymphocytes were lysed with RLT buffer (QIAGEN, Venlo, the Netherlands) as described in the QIAGEN RNAeasy kit protocol and stored at –80°C until use.

*EROD activity*. We determined hepatic CYP1A1 activity using ethoxyresorufin-*O*-deethylase (EROD) activity in hepatic microsomal fractions as described by Schulz et al. (2012).

*RNA isolation and quantitative real-time polymerase chain reaction (PCR)*. Total RNA was isolated from liver and PBLs using a QIAGEN RNeasy kit (QIAGEN). Purity and concentration of the isolated RNA was determined by measuring the absorbance ratio at 260/280 nm and 230/260 nm with a Nanodrop 2000 spectrophotometer (Thermo Scientific, Asheville, NC, USA). RNA was reverse transcribed to complementary DNA (cDNA) using the iScript cDNA synthesis Kit (Bio-Rad, Veenendaal, the Netherlands). Quantitative real-time PCR analyses were performed using the iQ Real-Time PCR Detection System with SYBR green (Bio-Rad). Amplification reactions were set up with 15 μL mastermix containing 12.5 μL iQ SYBR Green Supermix (Bio-rad), 0.5 μL distilled H_2_O, 1 μL (10 μM) forward primer, 1 μL (10 μM) reverse primer, and 10 μL first strand cDNA (10X diluted). Primer sequences were as follows: *Cyp1a1*: forward-5´-GGTT​AACC​ATGA​CCGG​GAAC​T-3´ and reverse-5´-TGCC​CAAA​CCAA​AGAG​AGTG​A-3´ (Schulz et al. 2012); *Cyp1a2*: forward-5´-ACATT​CCCA​AGGA​GCGC​TGTA​TCT-3´ and reverse-5´-GTCG​ATGG​CCGA​GTTG​TTAT​TGGT-3´ (Flaveny et al. 2010); *Cyp1b1*: forward-5´-GTGG​CTGC​TCAT​CCTC​TTTA​CC-3´ and reverse-5´-CCCA​CAAC​CTGG​TCCA​ACTC-3´ (Berge et al. 2004); β*-actin*: forward-5´-ATGC​TCCC​CGGG​CTGT​AT-3´ and reverse-5´-CATA​GGAG​TCCT​TCTG​ACCC​ATTC-3´ (Schulz et al. 2012). All primers were run through the National Center for Biotechnology Information Primer-BLAST database (http://www.ncbi.nlm.nih.gov/tools/primer-blast/) to confirm specificity and validate for optimal annealing temperature (60°C for all primers) and efficiency. The efficiency of all primer pairs was 98–102% (tested at 60°C). The following program was used for denaturation and amplification of cDNA: 3 min at 95°C, followed by 40 cycles of 15 sec at 95°C and 45 sec at 60°C. Gene expression for each sample was expressed as threshold cycle (*C*_t_), normalized to the reference gene β-*actin* (Δ*C*_t_). We calculated fold induction relative to the control group.

*Data analysis*. We obtained concentration–response curves using a sigmoidal dose–response nonlinear regression curve fit with variable slope (GraphPad Prism 6.01; GraphPad Software Inc., San Diego, CA, USA):


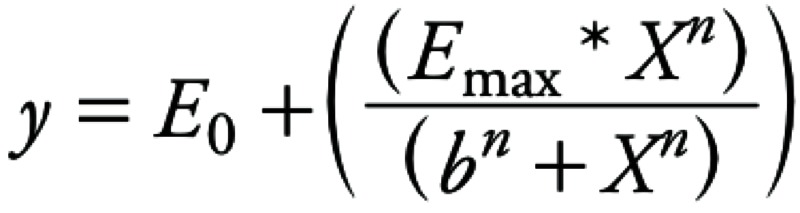
[1]

In this Hill equation, *y* is the dependent variable (EROD activity or fold induction of mRNA levels), *x* the independent variable (administered or systemic dose), *E_0_* is the estimated background response level, *E_max_* is the maximum response, *b* is the estimated median effective concentration (EC_50_), and *n* is the shaping parameter of the Hill curve.

We calculated the potency of a congener relative to TCDD using the dose or concentration [benchmark response (BMR)] needed for a congener to reach 20% of the TCDD response (BMR_20TCDD_). Using the congener-specific BMR_20TCDD_ concentration, REPs were calculated relatively to TCDD:


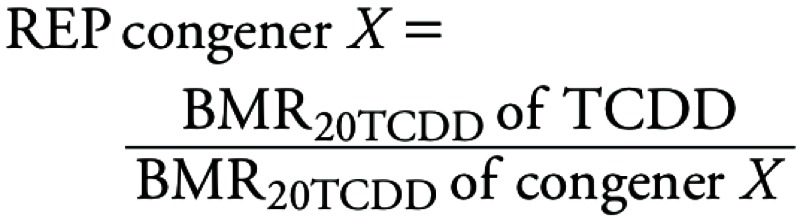
[2]

*Statistical analysis*. Statistical significant differences of the means and variances were determined using one-way analysis of variance (ANOVA) followed by Tukey-Kramer multiple comparisons. Differences were considered statistically significant at *p* < 0.05. Statistical calculations were performed using GraphPad 6.01 (GraphPad Software Inc.).

## Results

*Effect on body and organ weight*. To evaluate the possible toxic effects of the congeners tested, we examined body and organ weights. We observed no changes in body weight in congener-treated mice compared with vehicle controls. Relative thymus weights showed a decreasing trend for all compounds except PCB-126; however, this decrease was statistically significantly different from the vehicle controls only in mice treated with TCDD (≥ 2.5 μg/kg BW), PeCDD (0.5, 10, and 100 μg/kg BW), and PCB-153 (500,000 µg/kg BW). We also observed a dose-dependent increasing trend in liver weight for all compounds, but this increase was significantly different from vehicle controls only at doses of ≥ 10 µg/kg BW (TCDD), ≥ 100 µg/kg BW (PeCDD), ≥ 100 µg/kg BW (4-PeCDF), ≥ 1,000 µg/kg BW (PCB-126), ≥ 150,000 µg/kg BW (PCB-118), ≥ 50,000 µg/kg BW (PCB-156), and ≥ 500,000 μg/kg BW (PCB-153). In addition, we observed a dose-dependent increasing trend in hepatic lipid content of mice treated with all compounds except PCB-153, compared with vehicle controls. No statistically significant changes in spleen weight were observed for any of the compounds tested. Additional information is provided in Supplemental Material, Table S2 (http://dx.doi.org/10.1289/ehp.1206336).

**Figure 1:**
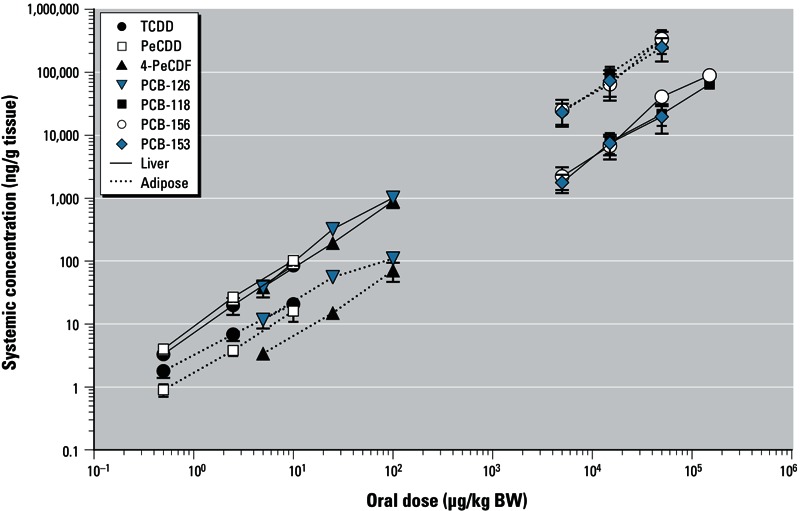
Relationship between oral dose and mean systemic concentration in liver or adipose tissue of female C57BL/6 mice 3 days after administration of a single dose of TCDD, PeCDD, 4-PeCDF, PCB‑126, PCB‑118, PCB‑156, or PCB‑153. Data represent mean ± SD of 6 mice.

*Distribution of the compounds*. To calculate ^systemic^REPs, we analyzed liver, adipose, and plasma concentrations of the test compounds [see Supplemental Material, Table S3 (http://dx.doi.org/10.1289/ehp.1206336)]. Within the 3-day period between dosing and sacrifice, concentrations of all congeners increased linearly with the administered dose (Figure 1), which indicates an absence of autoinduction of metabolism for the different dose levels within this time period.

On a wet weight basis (nanograms per gram of tissue), concentrations of TCDD, PeCDD, 4-PeCDF, and PCB-126 were higher in the liver than in adipose tissue [see Supplemental Material, Table S3 (http://dx.doi.org/10.1289/ehp.1206336)]. In contrast, concentrations of the mono*-ortho* PCBs 156 and 118, and the non-dioxin-like PCB-153 were lower in liver than in adipose tissue. These differences were even more pronounced when concentrations were expressed as percent of dose per gram of tissue. Thus, the more potent DLCs had a higher liver affinity than the less potent PCBs 118 and 156. Therefore, we determined the ratio between liver and adipose tissue concentrations to study congener-specific hepatic sequestration. Diliberto et al. (1997) previously suggested that a liver:adipose ratio > 0.3 reflects congener-specific hepatic sequestration. In our study, we observed liver:adipose ratios > 0.3 for TCDD, PeCDD, 4-PeCDF, and PCB-126 but liver:adipose ratios < 0.3 for PCBs 118, 156, and 153 (Table 1). Hepatic sequestration was dose dependent for TCDD and PCB-126 (as shown by increasing liver:adipose ratios at higher dose levels) but not for PeCDD and 4-PeCDF.

**Table 1:** Liver:adipose concentration ratios.

Congener	Dose (μg/kg BW)	Liver:adipose ratio
TCDD	0.5	1.8±0.2
2.5	2.9±0.5*
10	4.2±0.7*
PeCDD	0.5	4.4±0.9
2.5	7.0±1.1*
10	6.7±1.4
4-PeCDF	5	11.5±1.7
25	13.2±1.5
100	13.3±2.6
PCB-126	5	3.2±0.3
25	5.9±0.9*
100	9.1±0.9*
PCB-118	15,000	0.08±0.01
50,000	0.07±0.02
PCB-156	5,000	0.09±0.02
15,000	0.11±0.03
50,000	0.12±0.02
PCB-153	5,000	0.08±0.02
15,000	0.11±0.02
50,000	0.08±0.03
Data represent the mean±SD (based on ng/g tissue) of 6mice. *p<0.05 compared with the next lower dose, determined by one-way ANOVA followed by Tukey’s multiple comparisons test.		

*Dose–response curves*. We used tissue and plasma concentrations to determine dose–response relationships of hepatic EROD activity and gene expression of *Cyp1a1*, *1b1,* and *1a2* in liver and PBLs [see Supplemental Material, Figure S1 (http://dx.doi.org/10.1289/ehp.1206336)]. All compounds except PCB-153 caused a statistically significant, dose-dependent increase in hepatic EROD activity and in *Cyp1a1* and *1a2* mRNA levels. Hepatic *Cyp1b1* mRNA expression was dose-dependently increased by TCDD, PCDD, 4-PeCDF, and PCB-156. We observed a dose-dependent trend for PCB-118; however, the maximum induction for PCB-118 was < 0.3% of the maximal response of TCDD. PCB-126 did not induce *Cyp1b1* mRNA levels in the liver. In PBLs, *Cyp1a1* mRNA levels were dose-dependently induced by all compounds except PCB-118 and PCB-153. *Cyp1b1* mRNA was statically significantly and dose-dependently induced by TCDD, PeCDD, and 4-PeCDF. PCB-126 induced *Cyp1b1* mRNA only at the highest dose tested, with 3.5% of the maximal induction of TCDD. PCB-118, PCB-156, and PCB-153 did not induce *Cyp1b1* mRNA levels in PBLs, and *Cyp1a2* mRNA was not expressed in PBLs.

For all DLCs, a maximum induction (*Y*_max_) was reached only for hepatic EROD activity but not for *Cyp1a1*, *1b1,* or *1a2* mRNA in the liver and PBLs, even at the highest doses tested. Furthermore, we observed differences in curve Hill slopes between congeners for all end points tested [see Supplemental Material, Figure S1 (http://dx.doi.org/10.1289/ehp.1206336)]. Dose–response curves of *Cyp1a1* mRNA in liver and PBLs based on administered dose or on liver or plasma concentration are provided in Supplemental Material, Figure S2. Congener-specific differences in *Y*_max_ and Hill slopes can add a significant uncertainty in calculating EC_50_ values that generally form the basis of REP determination. To reduce this uncertainty, we focused on the lower part of the dose–response curves (BMR_20TCDD_) as a comparative end point (see Supplemental Material, Figures S1 and S2).

**Figure 2:**
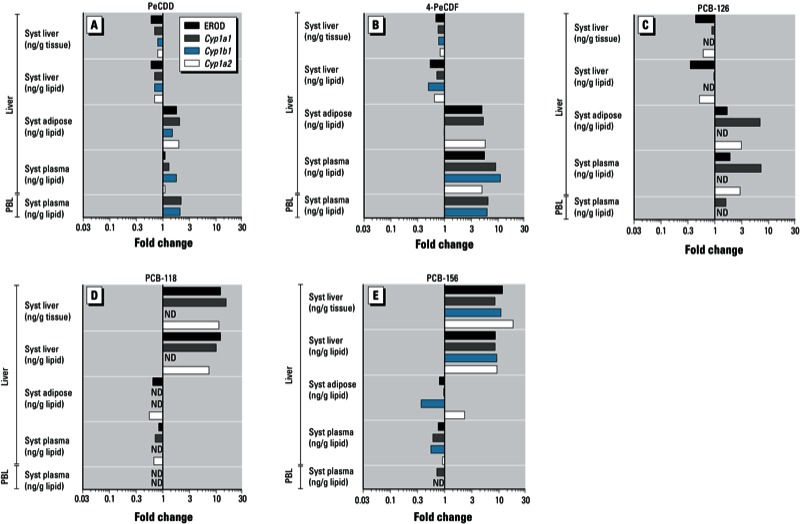
Fold change in ^systemic^REP compared with ^intake^REP (set to 1) for PeCDD (*A*), 4-PeCDF (*B*), PCB-126 (*C*), PCB-118 (*D*), and PCB-156 (*E*). Changes in REPs were calculated for hepatic EROD activity, and *Cyp1a1*, *Cyp1b1*, and *Cyp1a2* gene expression in liver and PBLs. Abbreviations: ND, not determined; Syst, systemic.

*BMR_20TCDD_ concentrations and REPs*. BMR_20TCDD_ values for hepatic end points were calculated based on administered dose and on hepatic, adipose, or plasma concentration, whereas BMR_20TCDD_ for PBL end points were calculated using only the administered dose or plasma concentration. The administered dose or systemic levels needed for a congener to reach the BMR_20TCDD_ varied strongly between end points, but also between the liver and PBLs (Table 2). Compared with liver, a higher concentration was usually needed in PBLs to reach a BMR_20TCDD_ for the same end point. In the liver, EROD activity was the most sensitive biomarker for TCDD, PeCDD, 4-PeCDF, and PCB-126 exposure, followed by *Cyp1a1* and *Cyp1a2* mRNA induction. In contrast, hepatic *Cyp1a2* mRNA induction appeared to be the most sensitive biomarker for PCB-118 and PCB-156, followed by EROD activity and *Cyp1a1* gene expression. In PBLs in the TCDD group, the BMR_20TCDD_ for *Cyp1a1* and *Cyp1b1* were similar. In contrast, for PeCDD and 4-PeCDF, the BMR_20TCDD_ of *Cyp1b1* expression was at least twice that of *Cyp1a1* gene expression. In Figure 2, we present an overview of the REP differences based on liver, adipose, and plasma concentrations. A BMR_20TCDD_ was not reached for all congeners or end points studied; thus, these data were excluded from the REP calculations.

**Table 2:** Mean BMR_20TCDD_ concentrations for TCDD, PeCDD, 4-PeCDF, PCB-126, PCB-118, and PCB-156 and corresponding REPs for various end points in liver and PBLs.

Tissue/biomarker	Dose metric	TCDD		PeCDD		4-PeCDF		PCB-126		PCB-118		PCB-156	
		BMR_20TCDD_	REP	BMR_20TCDD_	REP	BMR_20TCDD_	REP	BMR_20TCDD_	REP	BMR_20TCDD_	REP	BMR_20TCDD_	REP
LiverEROD activity	Adm dose (µg/kg BW)	0.29	1	0.54	0.5	4.11	0.07	29.3	0.01	55,259	0.000005	15,664	0.00002
Syst liver (ng/g liver)	1.61	1	4.85	0.3	32.9	0.05	373	0.004	25,441	0.00006	7,501	0.0002
Syst liver (ng/g lipid)	34.6	1	99.6	0.3	913	0.04	9,938	0.003	720,241	0.00006	217,711	0.0002
Syst adipose (ng/g lipid)	1.23	1	1.25	1	3.47	0.4	72.7	0.02	359,114	0.000003	82,483	0.00001
Syst plasma (ng/g lipid)	1.38	1	2.31	0.6	3.50	0.4	72.3	0.02	311,118	0.000004	98,188	0.00001
Liver*Cyp1a1 *mRNA	Adm dose (µg/kg BW)	0.64	1	1.25	0.5	81.3	0.008	558	0.001	139,631	0.000005	44,305	0.00001
Syst liver (ng/g liver)	4.35	1	12.0	0.4	725	0.006	4,299	0.001	62,418	0.00007	35,669	0.0001
Syst liver (ng/g lipid)	77.5	1	216	0.4	13,768	0.006	70,368	0.001	1,693,882	0.00005	634,215	0.0001
Syst adipose (ng/g lipid)	2.50	1	2.36	1	59.8	0.04	315	0.008	ND		180,515	0.00001
Syst plasma (ng/g lipid)	2.66	1	3.94	0.7	37.6	0.07	0.32	0.008	803,766	0.000003	303,586	0.000009
Liver*Cyp1b1 *mRNA	Adm dose (µg/kg BW)	3.55	1	10.1	0.4	150	0.02	ND		ND		95,664	0.00004
Syst liver (ng/g liver)	29.1	1	105	0.3	1,577	0.02	ND		ND		72445.7	0.0004
Syst liver (ng/g lipid)	391	1	1,655	0.2	32,921	0.01	ND		ND		1,158,251	0.0003
Syst adipose (ng/g lipid)	10.3	1	19.4	0.5	461	0.02	ND		ND		745,126	0.00001
Syst plasma (ng/g lipid)	11.6	1	18.5	0.6	44.6	0.3	ND		ND		553,459	0.00002
Liver*Cyp1a2 *mRNA	Adm dose (µg/kg BW)	0.41	1	0.56	0.7	8.83	0.05	87.4	0.005	15,522	0.00003	12,085	0.00003
Syst liver (ng/g liver)	2.59	1	4.59	0.6	68.1	0.04	912	0.003	8,833	0.0003	4,239	0.0006
Syst liver (ng/g lipid)	51.1	1	95.3	0.5	1,712	0.03	21,240	0.002	267,405	0.0002	166,060	0.0003
Syst adipose (ng/g lipid)	1.73	1	1.20	1	6.53	0.3	120	0.01	117,517	0.00001	22,134	0.00008
Syst plasma (ng/g lipid)	1.85	1	2.36	0.8	8.01	0.2	135	0.01	103,230	0.00002	60,702	0.00003
PBLs*Cyp1a1 *mRNA	Adm dose (µg/kg BW)	22.4	1	33.7	0.7	117	0.2	603	0.04	ND		747,734	0.00003
Syst plasma (ng/g lipid)	50.6	1	34.0	1.5	40.8	1	847	0.06	ND		2,359,081	0.00002
PBLs*Cyp1b1 *mRNA	Adm dose (µg/kg BW)	20.9	1	51.8	0.4	514	0.04	ND		ND		ND
Syst plasma (ng/g lipid)	53.5	1	63.8	0.8	212	0.3	ND		ND		ND
PBLs*Cyp1a2 *mRNA	Adm dose (µg/kg BW)	ND		ND		ND		ND		ND		ND
Syst plasma (ng/g lipid)	ND		ND		ND		ND		ND		ND
Abbreviations: Adm, administered; ND, not determined because BMR_20TCDD_ was not reached; Syst, systemic. Data are expressed as mean BMR_20TCDD_ derived from dose–response curves of 6 mice. REPs were calculated as described in “Materials and Methods.“													

For comparison of congener-specific REPs across exposure matrices (intake, liver, adipose, or plasma), the ^intake^REP was set to 1 and deviations were calculated for various ^systemic^REPs with the same end point (Figure 2). We observed two different types of deviations between ^systemic^REPs and ^intake^REPs. Based on liver concentrations (wet weight or lipid weight), ^systemic^REPs of PeCDD, 4-PCDF, and PCB-126 were at most one-third of the ^intake^REPs. In contrast, ^systemic^REPs of PCB-118 and PCB-156 are up to one order of magnitude higher than their ^intake^REPs. When ^systemic^REPs for hepatic effects of PeCDD, 4-PeCDF, and PCB126 were calculated using adipose tissue and plasma concentrations, ^systemic^REPs were up to one order of magnitude higher than ^intake^REPs, depending on the end point studied. We found the opposite for the ^systemic^REPs of PCB-118 and PCB-156, which were at most one-third of the ^intake^REPs. In PBLs, ^systemic^REPs based on plasma concentrations also deviated from ^intake^REPs, in a manner similar to that of ^systemic^REPs of hepatic end points based on plasma concentration.

These two different types of deviations from ^intake^REPs that we found for ^systemic^REPs differentiate the more potent AhR agonists (PeCDD, 4-PeCDF, and PCB-126) from the less potent mono*-ortho* PCBs (PCB-118 and PCB-156). In both groups, ^systemic^REPs can differ as much as one order of magnitude from the ^intake^REPs (Figure 2).

## Discussion

The TEF approach is the most commonly used method of assessing the risk of complex mixtures of dioxins and DLCs. Current TEF values are derived mainly from a range of ^intake^REPs, preferably from (sub)​chronic *in vivo* studies. These ^intake^REPs link the administered dose to a toxic or biological effect, subsequently leading to the derivation of ^intake^TEFs (Van den Berg et al. 1998, 2006).

At present, available data are insufficient to establish whether or not ^intake^TEFs are valid for risk assessment based on plasma or adipose tissue concentrations. Thus far, the limited experimental evidence available suggests that ^systemic^REPs of DLCs may differ from ^intake^REPs (Budinsky et al. 2006; DeVito et al. 1997, 2000). This discrepancy originates most likely from toxicokinetic differences between various DLCs. Several studies have shown that many DLCs bind strongly to CYP1A2 protein and, as a result, strongly sequester in the rodent liver (DeVito et al. 1998; Diliberto et al. 1995, 1997, 1999). This binding affinity toward CYP1A2 influences the hepatic, plasma, and adipose tissue disposition of DLCs. This was confirmed using CYP1A2 knockout mice in which the liver:adipose ratio decreased to < 0.3 for TCDD and 4-PeCDF, which is indicative of no hepatic sequestration (Diliberto et al. 1997). These ratios are significantly lower than those we observed in the present study for both congeners (Table 1). It is worth nothing that the dose dependency and hepatic sequestration we observed in our single dose, 3-day study are similar for all tested compounds—except for 4-PeCDF at the two highest concentrations tested—to those observed by DeVito et al. (1998) in a multiple dose, subchronic 13-week study of female B6C3F1 mice. In addition, the TCDD EC_50_ systemic liver concentrations for hepatic EROD activity were similar. Comparable findings can also be expected for the other DLCs tested because metabolism and elimination of these compounds are very similar. In light of the similarities between results of the two studies, we assume that ^intake^REPs and ^systemic^REPs do not deviate over time, even when they have not reached a steady state. In the present study, ^intake^REPs and ^systemic^REPs for *Cyp1a1*, *1a2*, and *1b1* induction were determined 3 days after a single oral dose. Previous studies have shown that hepatic CYP1A1, 1A2, and 1B1 protein levels are already maximal in rats 3 days after a single dose of TCDD (Santostefano et al. 1997). Although induction of CYP1A1, 1A2, and 1B1 enzymes is not a measure of toxicity, this is considered to be the most sensitive biomarker for AHR activation (Abel and Haarmann-Stemmann 2010; Denison and Heath-Pagliuso 1998). Moreover, studies have shown a high correlation in REPs between induction of these enzymes and toxic responses inflicted by DLCs, such as wasting syndrome, thymic atrophy, or hepatic porphyrin accumulation (Safe 1990; Van Birgelen et al. 1996).

**Figure 3:**
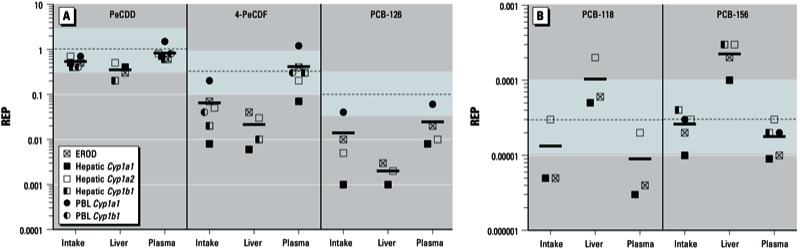
REPs determined in the present study in relation to the WHO-TEF ± half log uncertainty range. REPs were determined for EROD activity, and gene expression of *Cyp1a1*, *Cyp1b1*, and *Cyp1a2* in the liver, and gene expression of *Cyp1a1* and *Cyp1b1* in PBLs of mice administered a single dose of PeCDD, 4-PeCDF, or PCB‑126 (*A*) or of PCB‑118 and PCB‑156 (*B*). REPs for hepatic end points were calculated based on administered dose (Intake), lipid-based liver concentration (Liver), or lipid-based plasma concentration (Plasma); for PBLs, REPs were calculated using the administered dose or plasma concentration. Black lines represent the mean of the REPs; the dotted line and the blue shaded area represent the WHO-TEF ± half log uncertainty range.

Similar to earlier studies, we observed distinct deviations between ^intake^REPs and ^systemic^REPs based on liver, plasma, or adipose tissue concentrations (Budinsky et al. 2006; DeVito and Birnbaum 1995; DeVito et al. 1997, 2000). We observed congener-specific differences between the potent PeCDD, 4-PeCDF, and PCB-126 versus the less potent mono*-ortho* PCBs, PCB-118 and PCB-156 (Figure 2). On the basis of the liver:adipose ratios established in our study (Table 1), it appears that these congener-specific differences have a toxicokinetic basis, in which hepatic sequestration due to CYP1A2 binding plays a significant role. It is unclear whether a CYP1A2-sequestered compound is bioavailable to activate the AhR and cause dioxin-like responses. For this reason, REPs calculated on total hepatic tissue concentration, instead of the “free” available concentrations, may lead to either an over- or underestimation of the potency of a congener, depending on the relative degree of hepatic sequestration compared with that of TCDD. The ^systemic^REPs based on plasma concentrations for *Cyp1a1* and *1b1* gene expression in PBLs and liver show similar deviations from ^intake^REPs for all DLCs tested. The ^systemic^REPs are sometimes more than half a log unit different from the ^intake^REPs, which is more than the assumed uncertainty range applied to the WHO-TEF values (Van den Berg et al. 2006). To further address this issue, we compared ^intake^REPs and ^systemic^REPs from the present study with existing WHO-TEF values and the half log uncertainty around that value (Figure 3). On the basis of this comparison, we observed that

REPs of PeCDD fall mostly within the uncertainty range of the WHO-TEF of 1, with no large difference between ^systemic^REPs and ^intake^REPs.
Based on the intake dose and hepatic concentrations, deviations from the half log unit uncertainty are observed for 4-PeCDF, but ^systemic^REPs based on plasma concentrations are close to the WHO-TEF of 0.3.
For PCB-126, ^intake^REPs and ^systemic^REPs are up to two orders of magnitude below the WHO-TEF value of 0.1. Of all end points studied, only *Cyp1a1* mRNA expression in PBLs falls within the half log unit uncertainty.
REPs based on intake dose and plasma concentrations for mono-*ortho* PCBs 118 and 156 are consistently lower than the WHO-TEFs of 0.00003. In contrast, REPs based on liver effects and concentrations are significantly higher than the WHO-TEFs for both PCBs. However, because of differences in Cyp1a2 sequestration between the mono-*ortho* PCBs and the reference compound TCDD, caution should be taken not to overinterpret these liver-based ^systemic^REPs.

Most REPs determined in the present study are significantly lower than those established by the WHO (Van den Berg et al. 2006). However, the WHO-TEFs were derived from a range of ^intake^REPs often involving (semi)chronic studies and different species, whereas our study involves a single-dose exposure with relatively acute effects after 3 days only in mice. In the present study, we did not aim to recalculate or debate the current WHO-TEFs or methodology. However, the current WHO-TEF concept is based on the assumption that ^intake^REPs represent ^systemic^REPs, but a full data set to reject or accept this assumption is lacking. In our study, we compared ^intake^REPs with ^systemic^REPs obtained from a mouse model to provide more knowledge about possible deviations between both types of REPs. More data, for example, additional *in vivo* rat data and human *in vitro* data from our EU-SYSTEQ project studies, may provide additional information with respect to deviation of the ^intake^REPs and ^systemic^REPs from our studies with current WHO-TEF values. With these additional data, it can then be discussed whether ^systemic^REPs would better reflect a risk than intake (WHO-)TEFs.

## Conclusions

There are significant differences between ^intake^REPs and ^systemic^REPs for hepatic EROD activity and *Cyp1a1*, *1a2,* and *1b1* gene expression in the liver and PBLs. To avoid flawed calculations due to, for example, congener-specific hepatic sequestration, it may be more appropriate to use blood or adipose tissue as a matrix to calculate ^systemic^REPs. The ^systemic^REPs based on plasma/adipose concentration in our study are sometimes more than half a log unit different from the ^intake^REPs. This suggests that using ^intake^REPs or ^intake^TEFs to calculate TEQs in blood for PeCDD, 4-PeCDF, and PCB-126 result in an underestimation of the risk. In contrast, using ^intake^REPs or ^intake^TEFs for the mono*-ortho* PCBs 118 and 156 to calculate blood TEQs in blood may lead to an overestimation of the risk. Overall, our comparison of ^intake^REPs and ^systemic^REPs in mice reveals significant congener-specific differences that warrants the development of ^systemic^TEFs to calculate TEQs in blood and body tissues.

## Supplemental Material

(1.3 MB) PDF
