# Supplementary material for: Comparison of Intake and Systemic Relative Effect Potencies of Dioxin-like Compounds in Female Mice after a Single Oral Dose
Source: Environ Health Perspect. 2013 May 3;121(7):847–53. doi: 10.1289/ehp.1206336 (PMC3702004; doi:10.1289/ehp.1206336)
Supplement: (1.3 MB) PDF [file ehp.1206336.s001.pdf]

Supplemental Material
Comparison of Intake and Systemic Relative Effect Potencies of Dioxin-like Compounds in Female Mice after a Single Oral Dose

Karin I. van Ede, Patrik L. Andersson, Konrad PJ Gaisch, Martin van den Berg, and Majorie BM van Duursen

Table of Contents:

Table S1: Congeners, TEF-values and dose ranges

Table S2: Body weight, relative thymus, liver and spleen weights and % lipid/g liver

Table S3: PCDD / PCDF / PCBs concentrations in liver, adipose tissue and plasma 3 days after a single oral dose

Figure S1: Dose-response curves for EROD activity and gene expression of *Cyp1a1*, *Cyp1b1*, *Cyp1a2* in mouse liver and gene expression of *Cyp1a1* and *Cyp1b1* in peripheral blood lymphocytes (PBL) three days after a single oral dose of TCDD, PeCDD, 4-PeCDF, PCB-126, PCB-118 and PCB-156.

Figure S2: Dose-response curves for *Cyp1a1* gene expression in mouse liver and peripheral blood lymphocytes (PBL) three days after a single oral dose of TCDD, PeCDD, 4-PeCDF, PCB-126, PCB-118 and PCB-156 expressed using administered dose, plasma concentration or liver concentration.

Table S1: Congeners, TEF-values and dose ranges

Congener	TEF	Single oral dose (µg/kg bw)				
TCDD	1	0.5	2.5	10	25	100
PeCDD	1	0.5	2.5	10	25	100
4-PeCDF	0.3	5	25	100	250	1000
PCB-126	0.1	5	25	100	250	1000
PCB-118	0.00003	5000	15000	50000	150000	500000
PCB-156	0.00003	5000	15000	50000	150000	500000
PCB-153	ND	5000	15000	50000	150000	500000

Table S2: Body weight, relative thymus, liver and spleen weights and % lipid/g liver.

Congener	Oral dose µg/kg bw	Body weight (gram)[a]	Thymus % of bw[a]	Liver % of bw[a]	Spleen % of bw[a]	% lipid / g liver[b]
TCDD	0	19.3 ± 1.0	0.32 ± 0.03	4.88 ± 0.18	0.34 ± 0.03	4.2
	0.5	19.3 ± 0.8	0.28 ± 0.06	5.08 ± 0.27	0.34 ± 0.05	5.3
	2.5	19.2 ± 1.3	0.23 ± 0.05[c]	5.37 ± 0.20	0.32 ± 0.05	7.2
	10	18.8 ± 1.1	0.18 ± 0.06[c]	5.49 ± 0.37[c]	0.30 ± 0.04	7.4
	25	19.6 ± 0.8	0.23 ± 0.02[c]	5.86 ± 0.22[c]	0.34 ± 0.02	NA
	100	19.4 ± 0.6	0.19 ± 0.04[c]	6.09 ± 0.44[c]	0.34 ± 0.02	NA
PeCDD	0	19.3 ± 1.0	0.32 ± 0.03	4.88 ± 0.18	0.34 ± 0.03	4.2
	0.5	18.6 ± 1.3	0.21 ± 0.03[cd]	4.56 ± 0.34	0.30 ± 0.02	4.7
	2.5	19.4 ± 1.1	0.29 ± 0.04	5.02 ± 0.26	0.31 ± 0.03	6.0
	10	18.9 ± 1.2	0.23 ± 0.06[c]	5.32 ± 0.43	0.32 ± 0.04	6.2
	25	19.4 ± 1.5	0.26 ± 0.05	5.26 ± 0.27	0.32 ± 0.04	NA
	100	18.8 ± 1.1	0.24 ± 0.05[c]	5.83 ± 0.50[c]	0.33 ± 0.04	NA
4-PeCDF	0	20.0 ± 0.5	0.28 ± 0.07	5.01 ± 0.25	0.32 ± 0.03	3.1
	5	19.3 ± 1.2	0.26 ± 0.07	5.37 ± 0.32	0.29 ± 0.05	3.6
	25	19.0 ± 1.1	0.24 ± 0.04	5.32 ± 0.19	0.33 ± 0.04	4.5
	100	19.2 ± 1.0	0.22 ± 0.04	5.94 ± 0.27[cd]	0.32 ± 0.03	4.5
	250	19.8 ± 1.0	0.23 ± 0.03	6.05 ± 0.42[c]	0.34 ± 0.04	NA
	1000	20.2 ± 1.1	0.22 ± 0.04	6.35 ± 0.30[c]	0.32 ± 0.02	NA
PCB-126	0	20.0 ± 0.5	0.28 ± 0.07	5.01 ± 0.25	0.32 ± 0.03	3.1
	5	19.3 ± 0.9	0.27 ± 0.06	4.97 ± 0.38	0.33 ± 0.04	3.4
	25	18.9 ± 1.4	0.29 ± 0.05	4.96 ± 0.35	0.33 ± 0.06	3.7
	100	19.5 ± 0.7	0.25 ± 0.06	5.07 ± 0.19	0.31 ± 0.04	4.4
	250	19.3 ± 1.3	0.26 ± 0.04	5.43 ± 0.19	0.32 ± 0.02	NA
	1000	18.8 ± 0.9	0.27 ± 0.04	5.64 ± 0.20[c]	0.39 ± 0.16	NA
PCB-118	0	19.4 ± 0.9	0.26 ± 0.07	5.15 ± 0.30	0.31 ± 0.05	2.7
	5000	18.3 ± 0.9	0.27 ± 0.03	5.04 ± 0.22	0.32 ± 0.03	2.7
	15000	18.3 ± 0.8	0.23 ± 0.04	5.06 ± 0.22	0.33 ± 0.05	3.3
	50000	18.0 ± 1.8	0.20 ± 0.05	4.87 ± 0.41	0.30 ± 0.03	3.5
	150000	19.2 ± 0.8	0.21 ± 0.05	6.14 ± 0.45[cd]	0.34 ± 0.04	4.6
	500000	19.1 ± 1.0	0.23 ± 0.04	7.23 ± 0.29[cd]	0.31 ± 0.03	NA
PCB-156	0	19.4 ± 0.9	0.26 ± 0.07	5.15 ± 0.30	0.31 ± 0.05	2.7
	5000	17.7 ± 0.7	0.21 ± 0.05	4.71 ± 0.43	0.27 ± 0.03	3.0
	15000	18.7 ± 1.3	0.23 ± 0.04	5.14 ± 0.41	0.30 ± 0.03	3.3
	50000	18.0 ± 0.9	0.25 ± 0.02	5.96 ± 0.62[cd]	0.31 ± 0.04	5.7
	150000	19.2 ± 1.1	0.21 ± 0.04	6.79 ± 0.51[cd]	0.29 ± 0.02	6.8
	500000	18.7 ± 0.6	0.18 ± 0.05	8.10 ± 0.17[cd]	0.33 ± 0.03	NA
PCB-153	0	19.7 ± 0.8	0.31 ± 0.05	5.11 ± 0.27	0.33 ± 0.04	3.7
	5000	18.8 ± 1.1	0.25 ± 0.05	4.71 ± 0.34	0.30 ± 0.04	3.3
	15000	19.1 ± 0.5	0.26 ± 0.04	4.96 ± 0.10	0.36 ± 0.04	3.9
	50000	19.6 ± 0.8	0.29 ± 0.03	5.15 ± 0.38	0.33 ± 0.04	3.4
	150000	19.9 ± 1.1	0.25 ± 0.04	5.47 ± 0.37	0.34 ± 0.05	NA
	500000	18.8 ± 0.7	0.24 ± 0.04[c]	5.80 ± 0.30[c]	0.33 ± 0.05	NA

[a]Data represents the mean ± SD of 6 mice

[b]Data represents the % lipid per gram of pooled liver samples from 6 mice

Statistically significant changes were determined by one-way ANOVA analysis followed by a Tukey's multiple comparisons test, [c]Significantly different from control group (p<0.05).

[d]Significantly different from previous concentration (p<0.05)

NA = not analysed

Table S3: PCDD / PCDF / PCBs concentration in liver, adipose tissue and plasma 3 days after a single oral dose

Congener	Oral dose	Liver			Adipose			Plasma		
	µg/kg bw	ng/g tissue[a]	ng/g lipid[a]	% dose / g tissue	ng/g tissue[a]	ng/g lipid[a]	% dose / g tissue	ng/g tissue[b]	ng/g lipid[b]	% dose / g tissue
TCDD	0.5	3.3 ± 0.6	62 ± 12	33	1.8 ± 0.4	2.1 ± 0.5	18	0,0040[c]	2,2[c]	0.040
	2.5	20 ± 6	277 ± 83	40	6.9 ± 1.5	7.6 ± 1.7	14	0,0134[c]	8,4[c]	0.027
	10	85 ± 13	1152 ± 176	43	21 ± 3	24 ± 4	10	0.053 ± 0,008	35 ± 6	0.027
	25	NA			NA			0.108 ± 0,005	60 ± 3	0.022
	100	NA			NA			0.432 ± 0,054	240 ± 30	0.022
PeCDD	0.5	4.0 ± 0.7	85 ± 14	40	0.9 ± 0.2	1.1 ± 0.2	9.3	0,0037[c]	2,2[c]	0.037
	2.5	27 ± 6	446 ± 96	54	3.8 ± 0.7	4.3 ± 0.8	7.7	0,0120[c]	6,3[c]	0.024
	10	103 ± 23	1669 ± 378	51	16 ± 5	19 ± 6	8.0	0.032 ± 0,011	19 ± 7	0.016
	25	NA			NA			0.052 ± 0,004	30 ± 2	0.010
	100	NA			NA			0.298 ± 0,039	157 ± 21	0.015
4-PeCDF	5	39 ± 6	1067 ± 178	39	3.4 ± 0.7	4.0 ± 0.8	3.4	0,0082[c]	4,1[c]	0.008
	25	195 ± 27	4295 ± 603	39	15 ± 3	17 ± 3	3.0	0,0192[c]	20[c]	0.004
	100	902 ± 204	20037 ± 4531	45	71 ± 24	85 ± 29	3.5	0.074 ± 0,018	41 ± 10	0.004
	250	NA			NA			0.173 ± 0,023	87 ± 11	0.003
	1000	NA			NA			1.108 ± 0,067	583 ± 35	0.006
PCB-126	5	38 ± 11	1119 ± 334	38	12 ± 3	14 ± 4	12	0,0351[c]	27[c]	0.035
	25	323 ± 49	8858 ± 1350	65	56 ± 11	67 ± 13	11	0,0924[c]	66[c]	0.018
	100	1012 ± 217	23203 ± 4986	51	110 ± 19	127 ± 22	5.5	0.249 ± 0,026	147 ± 15	0.012
	250	NA			NA			0.654 ± 0,091	363 ± 50	0.013
	1000	NA			NA			2.773 ± 0,721	1733 ± 451	0.014
PCB-118	5000[d]	-	-	-	-	-	-	-	-	-
	15000	7950 ± 2870	242378 ± 87490	2.6	95790 ± 26799	103000 ± 28817	32	161 ± 15	100333 ± 9504	0.054
	50000	21667 ± 7554	622605 ± 217076	2.2	333750 ± 137042	375000 ± 153981	33	672 ± 157	280000 ± 65574	0.067
	150000	64500 ± 10055	1853448 ± 288932	2.2	NA			1869 ± 383	890000 ± 182483	0.062
	500000	NA			NA			5000 ± 721	2500000 ± 360555	0.05
PCB-156	5000	2233 ± 880	75450 ± 29735	2.2	25217 ± 11490	28333 ± 12910	25	79 ± 15	46667 ± 9074	0.079
	15000	6800 ± 2656	203593 ± 79530	2.3	65017 ± 29435	69167 ± 31314	22	182 ± 68	91000 ± 34395	0.061
	50000	41000 ± 9960	718039 ± 174429	4.1	339300 ± 102498	390000 ± 117813	34	659 ± 147	346667 ± 77675	0.066
	150000	89500 ± 1786	1567426 ± 314993	3.0	NA			1338 ± 279	743333 ± 155027	0.045
	500000	NA			NA			3740 ± 779	2200000 ± 458258	0.037
PCB-153	5000	1783 ± 571	54872 ± 17559	1.8	23250 ± 8546	25833 ± 9496	23	75 ± 7	37333 ± 3512	0.075
	15000	7500 ± 2707	190840 ± 68881	2.5	74167 ± 33252	83333 ± 37361	25	155 ± 44	91000 ± 26211	0.052
	50000	19833 ± 9218	585054 ± 271910	2.0	249400 ± 100810	286667 ± 115873	25	595 ± 273	350000 ± 160935	0.060
	150000	NA			NA			1487 ± 291	743333 ± 145717	0.050
	500000	NA			NA			4180 ± 503	2200000 ± 264575	0.042

[a]Data represents the mean ± sd of 6 mice [b]Data represents the mean ± sd of 3 pooled blood plasma samples (1 plasma sample = plasma of 2 mice)

[c]Data represents the outcome of a pooled blood plasma sample from 6 mice [d]Analysis failed in the sampling procedure

NA = not analysed

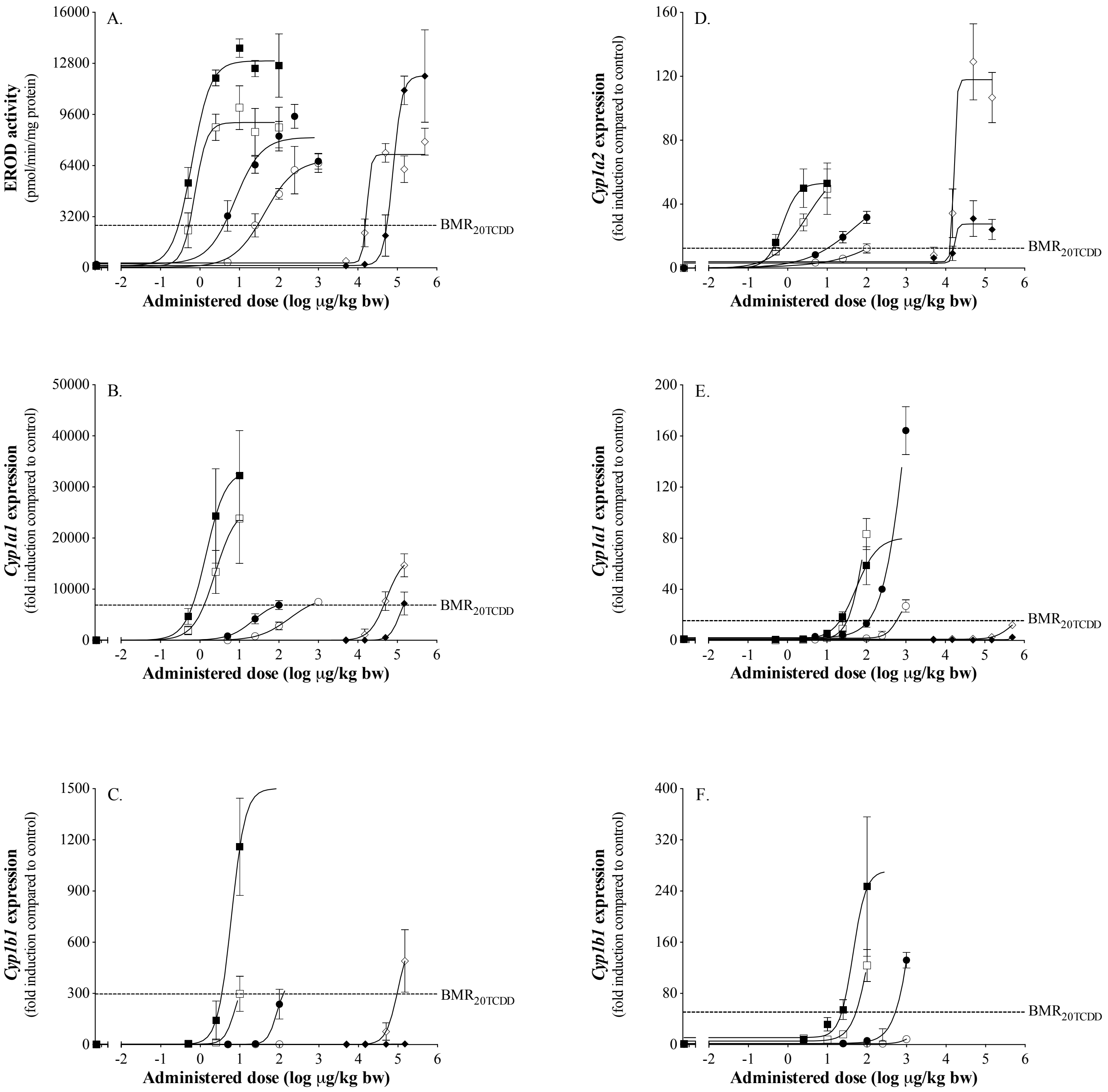

Figure S1: Dose-response curves for EROD activity (A) and gene expression of *Cyp1a1* (B), *Cyp1b1* (C), *Cyp1a2* (D) in mouse liver and gene expression of *Cyp1a1* (E) and *Cyp1b1* (F) in peripheral blood lymphocytes (PBL) three days after a single oral dose of TCDD (■), PeCDD (□), 4-PeCDF (●), PCB-126 (O), PCB-118 (◆) and PCB-156 (◇). Dose response curves are expressed using administered dose. BMR_{20TCDD} is indicated with a black dotted line. Data are represented as mean ± SD (n=6).

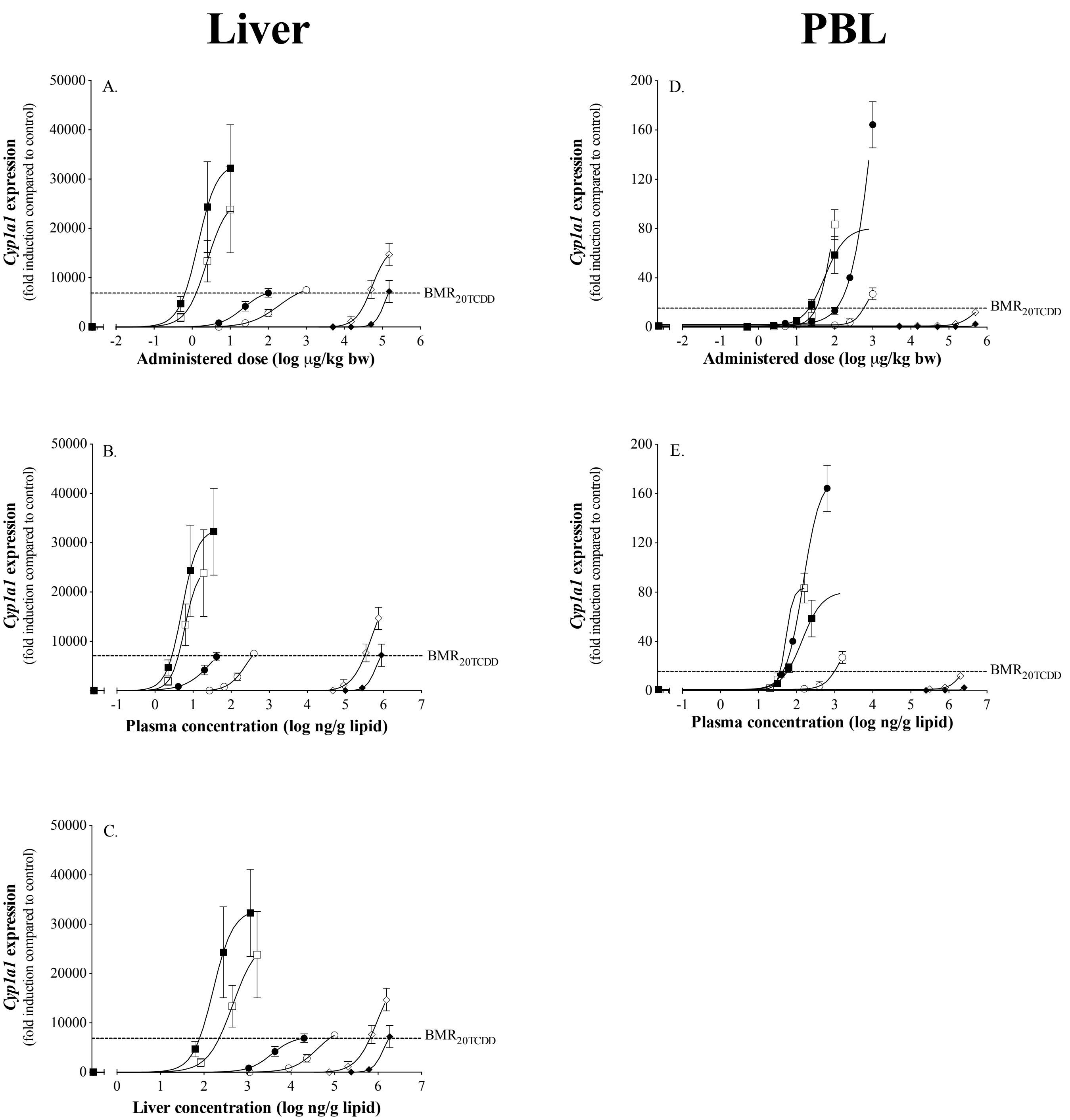

Figure S2: Dose-response curves of *Cyp1a1* gene expression in mouse liver and peripheral blood lymphocytes (PBL) three days after a single oral dose of TCDD (■), PeCDD (□), 4-PeCDF (●), PCB-126 (O), PCB-118 (◆) and PCB-156 (◇). Dose response curves are expressed using administered dose (A and D), plasma concentration (B and E) or liver concentration (C). BMR_{20TCDD} is indicated with a black dotted line. Data are represented as mean ± SD (n=6).
